# Potential Applications of Immobilized **β**-Galactosidase in Food Processing Industries

**DOI:** 10.4061/2010/473137

**Published:** 2010-12-27

**Authors:** Parmjit S. Panesar, Shweta Kumari, Reeba Panesar

**Affiliations:** Biotechnology Research Laboratory, Department of Food Engineering & Technology, Sant Longowal Institute of Engineering and Technology, Longowal, Punjab, 148 106, India

## Abstract

The enzyme **β**-galactosidase can be obtained from a wide variety of sources such as microorganisms, plants, and animals. The use of **β**-galactosidase for the hydrolysis of lactose in milk and whey is one of the promising enzymatic applications in food and dairy processing industries. The enzyme can be used in either soluble or immobilized forms but the soluble enzyme can be used only for batch processes and the immobilized form has the advantage of being used in batch wise as well as in continuous operation. Immobilization has been found to be convenient method to make enzyme thermostable and to prevent the loss of enzyme activity. This review has been focused on the different types of techniques used for the immobilization of **β**-galactosidase and its potential applications in food industry.

## 1. Introduction

The enzyme *β*-galactosidase (EC.3.2.1.23), most commonly known as lactase, which hydrolyses lactose into its monomers that is glucose and galactose has potential applications in food processing industry. Because of low levels of the enzyme in intestine, large fraction of the population shows lactose intolerance and they have difficulty in consuming milk and dairy products. Lactose has a low relative sweetness and solubility, and excessive lactose in large intestine can lead to tissue dehydration due to osmotic effects, poor calcium absorption due to low acidity, and fermentation of the lactose by microflora resulting in fermentative diarrhea, bloating, flatulence, blanching and cramps, and watery diarrhea [1]. Furthermore, lactose is a hygroscopic sugar and has a strong tendency to absorb flavours and odours and causes many defects in refrigerated foods such as crystallization in dairy foods, development of sandy or gritty texture, and deposit formation [2]. 

Technologically, lactose gets easily crystallized, which sets the limits of its applications to certain processes in the dairy industry. Cheese manufactured from hydrolyzed milk ripens more quickly than that made from normal milk. Treatment of milk and milk products with lactase to reduce their lactose content seems to be an appropriate method to increase their potential uses and to deal with the problems of lactose insolubility and lack of sweetness. Furthermore, this treatment could make milk, a most suitable food, available to a large number of adults and children that are lactose intolerant. Moreover, the hydrolysis of whey converts lactose into a very useful product like sweet syrup, which can be used in various processes of dairy, confectionary, baking, and soft drink industries [3, 4]. Therefore, lactose hydrolysis not only allows the milk consumption by lactose intolerant population but can also solve the environmental problems linked with whey disposal [5–7].

The enzyme *β*-galactosidase can also be used in transglycosylation of lactose to synthesize galacto-oligosaccharides (GOSs). These were widely recognized as the nondigestible oligosaccharides, not hydrolyzed or absorbed in the upper intestinal tract, and they pass onto the colon where they are fermented selectively by beneficial intestinal bacteria. Besides their prebiotic effects, these GOSs have low cariogenicity, low caloric values, and low sweetness [8, 9]. GOSs occur naturally in trace amounts in breast milk, cow milk, honey, and a variety of fruits and vegetables [10]. As a result, increased production of GOS is useful. GOS can be readily manufactured by enzymatic transgalactosylation of *β*-galactosidase from whey lactose, which is available in abundance as a by-product of cheese industry.

Thus, the application of *β*-galactosidase in the hydrolysis of lactose in dairy industry has attracted the attention of researchers. Although most industries still hydrolyze lactose with free enzyme, the immobilization of *β*-galactosidase is an area of great interest because of its potential benefits [11]. The use of immobilization technology is of significant importance from economic point of view since it makes reutilization of the enzyme and continuous operation possible and also precludes the need to separate the cells from the whey following processing. It can also help to improve the enzyme stability. Nowadays, immobilized *β*-galactosidase is intensively being used in lactose hydrolysis of milk/whey and has been tested for the production of galacto-oligosaccharides.

## 2. Microbial Sources of Enzyme

The enzyme *β*-galactosidase can be obtained from a wide variety of sources such as microorganisms, plants, and animals; however, according to their sources, their properties differ markedly [11, 12]. Enzymes of plants and animal origin have little commercial value but several microbial sources of *β*-galactosidase are of great technological interest. Microorganisms offer various advantages over other available sources such as easy handling, higher multiplication rate, and high production yield. As a result of commercial interest in *β*-galactosidase, a large number of microorganisms [13–26] have been assessed as potential sources of this enzyme (Table 1).

### 2.1. Production and Purification

Microorganisms are considered potential source of *β*-galactosidase for industrial applications. However, they differ in their optimum conditions for the enzyme application especially pH range. A recovery cost of the enzyme depends on the level of production and purification. Therefore, there has been increasing interest in finding microorganisms with adequate properties for industrial use, higher production capacity, and less expensive purification methods of this enzyme. A wide variety of bacterial, yeast, and fungal cultures have been reported for *β*-galactosidase production.

#### 2.1.1. Bacterial Enzymes

The enzyme *β*-galactosidase can be produced by a large number of bacteria but *Streptococcus thermophilus* and *Bacillus stearothermophilus* are considered as potential bacterial sources. The enzyme from *Escherichia coli *serves as a model for understanding the catalytic mechanism of *β*-galactosidase action, but it is not considered suitable for use in foods due to toxicity problems associated with the host coliform [11]. Hence, the *β*-galactosidase from *E. coli* is generally not preferred for use in food industry [13–15].

*β*-galactosidase has been isolated from an extremely thermophilic Gram-negative anaerobe. Thermoanaerobacter has been purified by chromatography through DEAE-cellulose [27]. The optimization of the ultrasonication methods for the maximum cell disruption of *Escherichia coli* for the release of *β*-galactosidase has also been reported [28]. *Lactobacillus delbrueckii *subsp. *bulgaricus* cultures were subjected to treatments using sonication, a high-speed bead mill, and a high-pressure homogenizer for the release of *β*-galactosidase [29]. 

*β*-galactosidase has also been purified from psychotropic *Pseudoalteromonas *sp. isolated from Antarctica and a high yield of purification has been reported by a rapid purification scheme using extraction in an aqueous two-phase system followed by hydrophobic interaction chromatography and ultrafilteration techniques [30].

An intracellular *β*-galactosidase from a thermoacidophilic *Alicyclobacillus acidocaldarius* subsp. *rittmannii* has been purified using precipitation (with ammonium sulphate), gel permeation, ion-exchange, and affinity chromatography and preparative electrophoresis [26]. Further, a thermostable **β**-galactosidase gene bgaB from *Bacillus stearothermophilus* was cloned and expressed in *B. subtilis* WB600 and recombinant enzyme has been purified by a combination of heat treatment, ammonium sulfate fractionation, ion exchange, and gel filtration chromatography techniques [31]. The intracellular *β*-galactosidase from thermophile B1.2 was purified by ion-exchange and affinity chromatography with a fold purification of 2.2 and 3.9, respectively. The molecular mass of the purified enzyme as determined by native PAGE was approximately 215 kDa, by SDS-PAGE was 75 kDa, and by gel filtration was 215 kDa [32]. The efficiency of different cell disruption methods on the extraction of intracellular *β*-galactosidase enzyme from *Streptococcus thermophilus* and* Lactobacillus delbrueckii* subsp*. thermophilus* has been tested [33]. Lysozyme enzyme treatment was determined as the most effective method, which resulted in approximately 1.5 and 10 times higher enzyme activity than glass bead and homogenization treatment, respectively.

 The activity and the stability of the partially purified *β*-galactosidases from *Thermus* sp strain T2 and *K. fragilis* have been compared [34]. Both enzymes showed a remarkable hydrolytic activity and a weak transgalactosilation activity, even in the presence of high concentrations of lactose. The thermophilic enzyme showed a higher resistance to hydrophobic agents and a higher stability at different temperatures, pHs, and chemical conditions. However, the enzyme of *Thermus* was less stable in the presence of oxygen peroxide, showing that some residues important for its stability were affected by oxidation. The enzyme from *K. fragilis* was strongly inhibited by o-nitrophenol in a acompetitive way but it was weakly and competitively inhibited by galactose. The thermophilic enzyme was competitively inhibited by galactose much strongly than its mesophilic counterpart but the inhibition did not change with the temperature. A novel thermostable chimeric *β*-galactosidase was constructed by fusing a poly-His tag to the N-terminal region of the *β*-galactosidase from *Thermus* sp. strain T2 to facilitate its overexpression in *E. coli* and its purification by immobilized metal-ion affinity chromatography [35]. To improve the enzyme purification a selective one-point adsorption was achieved by designing tailor-made low-activated Co-iminodiacetic acid (Co-IDA) or Ni-IDA supports. The new enzyme was not only useful for industrial purposes but also has become an excellent model to study the purification of large multimeric proteins via selective adsorption on tailor-made immobilized metal-ion affinity chromatography supports. Furthermore, *β*-galactosidase from *Thermus* sp. Strain T2 has been purified and immobilized in a single step, combining the excellent properties of epoxy groups for enzyme immobilization with the good performance of immobilized metal-chelate affinity chromatography for protein purification [36].

#### 2.1.2. Fungal Enzymes

The optimum pH range for the fungal enzyme is 2.5–5.4, which makes them suitable for processing of acid whey and its ultrafiltration permeate. The optimum temperatures for these enzymes are high and can be typically used at temperatures up to 50°C. The purification of *β*-galactosidase from different fungal sources has been carried using a variety of purification techniques.

*β*-galactosidase from *Aspergillus niger* has been purified and resolved into three multiple forms, using molecular sieving, ion exchange, and hydrophobic chromatography [37]. The purification of *β*-galactosidase has been carried from a cellular extract of *Fusarium oxysporum* var. *lini* by heat shock and successive chromatography on DEAE-cellulose DE-52 and sephadex G-100 [18]. The purification of *β*-galactosidase by ammonium sulphate precipitation and CM-sephadex chromatography from cell-free extracts of fungus *Beauveria bassiana *has also been reported [38]. An extracellular *β*-galactosidase from a themophilic fungus *Rhizomucor *sp. has been purified by successive DEAE-cellulose chromatography, followed by gel filtration on sephacryl S-300 [39]. The precipitation with ammonium sulfate, ion-exchange chromatography on DEAE-sephadex, affinity chromatography and chromatofocusing has also been used for purification of *β*-galactosidase from *Penicillium chrysogenum* NCAIM 00237 [40].

*β*-galactosidase produced by submerged culture of *Aspergillus japonicus *showed 2.95 U mg^−1^ protein specific activities with an approximate molecular weight of 27 kDa [41]. The enzyme was purified 6.43-fold with 24.02% yield and a specific activity of 18.96 U mg^−1^ protein. An intracellular *β*-glycoside hydrolase with *β*-glucosidase and *β*-galactosidase activity designated *β*-glucosidase BGL1 has been purified from the thermophilic fungus *Talaromyces thermophilus*. The monomeric enzyme has a molecular mass of 50 kDa (SDS-PAGE), an isoelectric point of 4.5-4.6. *β*-galactosidase activity of *β*-glucosidase BGL1 is activated by various mono and divalent cations including Na^+^, K^+^, and Mg^2+^, and it is moderately inhibited by its reaction products that is glucose and galactose [42].

#### 2.1.3. Yeast Enzymes

Yeast has been considered as an important source of *β*-galactosidase from industrial point of view. With neutral pH optima, these are well suited for hydrolysis of lactose in milk and are widely accepted as safe for use in foods. Much work has been carried on the production of *β*-galactosidase from different yeast strains for its potential use. The most commercially available yeast *β*-galactosidase under the trade name of *Maxilact* (DSM Food Specialties, Delft, The Netherlands) and *Lactase* (SNAM Progetti, Italy) is preparations extracted from *K. lactis *and* Lactozym* (Novo, Nordisk A/S, Bagsvaerd, Denmark) from *K. fragilis *[11, 14, 17].

Biermann and Glantz [43] first attempted the purification of *β*-galactosidase from *Sacharomyces lactis* by gel filtration on sephadex G-100, followed by ion exchange chromatography on DEAE-sephadex. The homogenizer (800 bar pressure, pH 7.5) was used for the disruption and extraction of *β*-galactosidase of *K. marxianus* [44]. Further, the partial purification of the enzyme from *K. marxianus* was carried using DEAE-sepharose column. The studies on the use of fed batch culture techniques to achieve high culture productivity in *K. fragilis* have also been carried out [45]. 

The optimization of *β*-galactosidase production by *K. lactis* using deprotienized whey as fermentation medium has been reported. The optimized condition for the enzyme production was reported as follows: temperature 30.3°C, pH 4.68, agitation speed 191 rpm, and fermentation time 18.5 hours [46]. The studies on two commercial *β*-galactosidase (Lactozym and Maxilact) preparations indicated that the enzyme activities of both enzyme preparations present similar behaviour with different pH and temperature and similar kinetic parameter values, which suggest that both enzymes are probably the same [47]. Response surface methodology has also been applied in the production of *β*-galactosidase using *K. lactis *NRRL Y-8279 [48] and maximum specific enzyme activity of 4218.4 U g^−1^was obtained at the optimum levels of process variables (pH 7.35, agitation speed 179.2 rpm, initial sugar concentration 24.9 g L^−1^, and incubation time 50.9 hours).

## 3. Immobilization of *β*-Galactosidase

Although the enzyme *β*-galactosidase has numerous applications in the food and dairy industries, but the moderate stability of enzyme is one of the limitations that hinder general implementation of biocatalysts at industrial scale. Thus, there is a need to explore their full potential as catalyst by adopting suitable strategies for enzyme stabilization. The multimeric enzyme can be stabilized by using proper experimental conditions and genetic tools to cross link or to strengthen the subunit-subunit interaction [49]. The stability of monomeric or multimeric enzymes can also be enhanced by multipoint and multi-subunit covalent immobilization and enzyme engineering via immobilization [50]. The enzyme has been immobilized by various methods such as physical absorption, entrapment, and covalent binding method [51–85] on different supports (Table 2).

### 3.1. Physical Adsorption

Physical adsorption is considered as the simplest method of immobilization in which an enzyme is immobilized onto a water-insoluble carrier and the biocatalysts are held on the surface of the carriers by physical forces (van der waals forces). Frequently, however, additional forces are involved in the interaction between carrier and biocatalyst principally hydrophobic interactions, hydrogen bridges, and heteropolar (ionic) bonds [86]. This method has the advantage of being simple to carry out and has little influence on the conformation of the biocatalyst. However, the disadvantage of this technique is the relative weakness of the adsorptive binding forces. Different inorganic (alumina, silica, porous glass, ceramics, diatomaceous earth, clay, bentonite, etc.) and organic (cellulose, starch, activated carbon and ion-exchange resins, such as Amberlite, Sephadex, Dowex) support materials can be used for enzyme adsorption. Further adsorption of enzyme may be stabilized by glutaraldehyde treatment. 

Immobilization of *β*-galactosidase on hydrophobic cotton cloth indicated that the enzyme adsorbed on the cloth was about 50% active as free enzymes [87]. The immobilization of *β*-galactosidase active yeast *K. fragilis *and* K. lactis* onto chitosan showed an enzyme activity of 0.9–2.2 U/mg dry cell wt [88]. Enzyme activity of immobilized enzyme from *K. fragilis* was higher but the operational stability of *A. oryzae* enzyme was 5–14 times higher depending upon the mode of immobilization [89]. When adsorption method was used, the highest activity was obtained with yeast enzyme and support Ostsorb-DEAE. The enzyme from *A. oryzae* immobilized on polyvinyl chloride (PVC) and silica gel membrane has been used for the hydrolysis of lactose in skim milk in an axial-annular flow reactor [51]. Further, maximum immobilization occurred at pH 5.5 and optimal results were obtained with citrate/phosphate buffer during immobilization of *β*-galactosidase from *E. coli* by physical adsorption on chromosorb-W [51]. A novel reactor consisting of *β*-galactosidase from *B. circulans* immobilized on a ribbed membrane composed of PVC and silica has also been used for skim milk lactose hydrolysis [53]. The immobilization of partially purified* Bullera singularisβ*-galactosidase in Chitopearl BCW 3510 bead (970 GU/g resin) by simple adsorption has also been carried out [90].

The studies on the kinetic behaviour of *β*-galactosidase from *Kluyveromces marxianus* (*Saccharomyces*) *lactis*, immobilized on to different oxide supports, such as alumina, silica, and silicated alumina indicated that the immobilized enzyme activity strongly depends on the chemical nature and physical structure of the support [53]. In particular, when the particle sizes of the support are increased, the enzymatic activity strongly decreases. Immobilization of *β*-galactosidase from *Thermus* sp. preceded very rapidly onto PEI-Sepabeads and conventional DEAE-Agarose. However, the adsorption strength was much higher in the case of PEI-Sepabeads [53]. 

A recombinant thermostable *B. stearothermophilusβ*-galactosidase was immobilized onto chitosan using Tris (hydroxymethyl) phosphine (THP) and glutaraldehyde, and a packed bed reactor was utilized to hydrolyze lactose in milk. The thermostability and enzyme activity of THP-immobilized *β*-galactosidase during storage was superior to that of free and glutaraldehyde-immobilized enzymes. The THP-immobilized *β*-galactosidase showed greater relative activity in the presence of Ca^2+^ than the free enzyme and was stable during the storage at 4°C for 6 weeks, whereas the free enzyme lost 31% of the initial activity under the same storage conditions [91]. Response surface methodology (RSM) and centre composite design (CCD) have been used to optimize immobilization of *β*-galactosidase (BGAL) from *Pisum sativum* onto two matrices: Sephadex G-75 and chitosan beads. The immobilization efficiency of 75.66% and 75.19% was achieved with Sephadex G-75 and chitosan, respectively [56]. 

*A. oryzaeβ*-galactosidase was immobilized on an inexpensive bioaffinity support, concanavalin A-cellulose. Concanavalin A-cellulose adsorbed and cross-linked *β*-galactosidase preparation retained 78% of the initial activity. The optimum temperature was increased from 50 to 60°C for the immobilized *β*-galactosidase. The cross-linked adsorbed enzyme retained 93% activity after 1-month storage while the native enzyme showed only 63% activity under similar incubation conditions [92].

### 3.2. Entrapment Method

Entrapment method is the physical enclosure of enzymes in a small space. Matrix and membrane entrapment (including microcapsulation) are the major methods of entrapment. The major advantage of the entrapment technique is the simplicity by which spherical particles can be obtained by dripping a polymer-cell suspension into a medium containing positively charged ions or through thermal polymerization [86]. Further, beads formed particularly from alginate are transparent and generally mechanically stable. The major limitation of this technique for the immobilization of enzymes is the possible slow leakage during continuous use in view of the small molecular size compared to the cells. However, improvements can be made by using suitable linking procedures. The matrices used for the immobilization are usually made up of polymeric materials such as Ca-alginate, agar, *k-*carragenin, polyacrylamide, and collagen. However, some solid matrices such as activated carbon, porous ceramic, and diatomaceous earth can also be used for the immobilization. The membranes commonly used for the entrapment of enzymes are nylon, cellulose, polysulfone, and polyacrylmide. 

Fungal *β*-galactosidase immobilized in polyvinyl alcohol gel was more thermostable than free enzyme and retained 70% of activity after 24 h at 50°C and 5% activity at 60°C [93]. The glutaraldehyde-treated *K. bulgaricus* cells having *β*-galactosidase were entrapped in alginate using BaCl_2_ solution [57]. The alginate beads obtained after treatment with polyethyleneimine followed by glutaraldehyde solution were stable. 

*E. coliβ*-galactosidase has been immobilized in polyacrylamide gels and through the preparation of cross-linked derivatives of *E. coliβ*-galactosidase by treating the enzyme with bisimidoesters. The combination of three protective agents, namely, bovine serum albumin, cysteine, and lactose, during immobilization gave an increased yield of 190% in the case of dimethyladipimidate (DMA) cross-linked preparation [58]. *K. marxianus* cells having lactase activity were entrapped in calcium pectate gel (CPG) and in calcium alginate gel (CAG) hardened by polyethyleneimine and glutaraldehyde. Permeabilized cells entrapped in CPG hydrolyzed lactose more than 80% in semicontinuous and continuous processes [94]. 

The comparison of the various methods of immobilization of *β*-galactosidase from *Thermus aquaticus* indicated that immobilization by cross-linking followed by entrapment in agarose beads can be beneficial for high enzyme loading with good activity yield [59]. The entrapment of *A. oryzae β*-galactosidase in a spongy polyvinyl alcohol cryogel increased the stability towards temperature, pH, and ionic strength more than the free enzyme [60]. The fibers composed of alginate and gelatin hardened with glutaraldehyde retained 56% relative activity of *β*-galactosidase for 35 days without any decrease. Moreover, the optimum conditions were also not affected by immobilization [95]. Another approach for immobilization of *β*-galactosidase is the use of liposomes and in this direction response surface methodology was applied to optimize the entrapment of the enzyme in liposomes by dehydration-rehydration vesicle method, which resulted in an entrapment efficiency of 28% [96]. 

It has been observed that entrapped cross-linked concanavalin A-*β*-galactosidase complex preparation was more superior in the continuous hydrolysis of lactose in a batch process as compared to the other entrapped preparations because it retained 95% activity after seventh repeated use and 93% of its original activity after 2-month storage at 4°C [97]. *A. oryzaeβ*-galactosidase was immobilized on the surface of a novel bioaffinity support: concanavalin A layered calcium alginate-starch beads. The maximum activity of the immobilized *β*-galactosidase has been obtained at 60°C, approximately 10 degrees higher than that of the free enzyme. It has been also observed that the immobilized *β*-galactosidase exhibited significantly higher stability to heat, urea, MgCl_2_, and CaCl_2_ than the free enzyme [98]. Calcium alginate-entrapped *β*-galactosidase preparations have been used for the hydrolysis of lactose from synthetic solution, milk, and whey in batch processes as well as in continuous packed bed columns. From the kinetic studies, it was observed that the Michaelis constant (*K*_*m*_) for the free and immobilized *β*-galactosidase was 2.51 mM and 5.18 mM, respectively. Moreover, the *V*_max_ for the soluble and immobilized enzyme was 4.8 × 10^−4^ mol/min and 4.2 × 10^−4^ mol/min, respectively [99]. 

The main problems associated with this type of immobilization process are desorption of *β*-galactosidase from immobilization matrix and the leakage of the entrapped enzyme due to a small molecular weight compared to pores of gel in matrices, which can be overcome by cross-linking using bifunctional or multifunctional reagents [100]. The conditions for polyethyleimine- (PEI-) coating of agarose supports to achieve a *β*-galactosidase derivative have been optimized that allows a high lactose conversion from whey in a steady bed-reactor with no enzyme leakage, together with good elution properties [101]. Various cross-linking reagents used for improvement of *β*-galactosidase stability in immobilized state are described in Table 3 [102–110].

### 3.3. Covalent Binding Method

Covalent binding is the retention of enzymes on support surface by covalent bond formation. Enzyme molecules bind to support material via certain functional groups such as amino, carboxyl, hydroxyl, and sulfydryl groups. These functional groups must not be in the active site. It is often advisable to carry out the immobilization in the presence of its substrate or a competitive inhibitor so as to protect the active site Functional groups on support material usually activated by using chemical reagents such as cyanogen bromide, carbodimide, and glutaraldehyde. 

Eggshells ground into pieces can be good carrier for immobilization of *β*-galactosidase because of its low cost, good mechanical strength, and resistance to microbial attack [61]. Fungal enzyme from *A. oryzae* has been immobilized onto powdered nylon-6 and zeolite [62]. Zeolites were nonideal since its coupling yield was low whereas nylon resulted in a stable matrix. The derivatives obtained either by diazo or by carbodiimide coupling showed the highest activities during immobilization of the enzyme on glycophase-coated porous glass [103]. 

*E. coliβ*-galactosidase immobilized onto gelatin using chromium (III) acetate and glutaraldehyde retained the relative activities of 25% and 22% for glutaraldehyde and chromium (III) acetate immobilized enzyme, respectively [64]. The enzyme immobilized on silica-alumina was more stable than the free form at acidic pH [65]. The ratio of protein to polymer also plays an important role during enzyme immobilization and 100% binding of protein to polymer can be obtained using optimal conditions [66]. 

The performance of immobilization of the thermostable *β*-galactosidase from *Thermus* sp. strain T2 on a standard Sepabeads-epoxy support with other Sepabeads-epoxy supports partially modified with boronate, iminodiacetic, metal chelates, and ethylenediamine was compared [109]. Immobilization yields depended on the support, ranging from 95% using Sepabeads-epoxy-chelate, Sepabeads-epoxy-amino, or Sepabeads-epoxy-boronic to 5% using Sepabeads-epoxy-IDA. The immobilized *β*-galactosidase derivatives showed very improved but different stabilities after favoring multipoint covalent attachment by long-term alkaline incubation, the enzyme immobilized on Sepabeads-epoxy-boronic being the most stable. The optimal derivative was very active in lactose hydrolysis even at 70°C, maintaining its activity after long incubation times under these conditions. Recently, the cross-linking of *β*-galactosidase on magnetic beads prepared from different sources (Artemisia seed gum, chitosan, and magnetic fluid) was done in the presence of glutaraldehyde and the effects of various preparation conditions on the activity of the immobilized *β*-galactosidase were studied. The immobilized *β*-galactosidase resulted in an increase in enzyme stability [110]. 

The heat stability of lactase can be increased through immobilization [66, 111, 112]. The effect of temperature and pH on the catalytic activity of immobilized *β*-galactosidase from *K. lactis* indicated that the temperature-activity curves are similar for both the free and immobilized enzymes [113]. However, the maximum activity of the immobilized enzyme was shifted from 40°C to 50°C compared with the free enzyme.

The comparison of a new and commercially available amino-epoxy support (amino-epoxy-Sepabeads) to conventional epoxy supports to immobilize *β*-galactosidase from *A. oryzae* showed that the enzyme stability can be significantly improved by the immobilization on this support, suggesting the promotion of some multipoint covalent attachment between the enzyme and the support [67]. The immobilization of thermophilic *β*-galactosidase on Sepabeads for lactose hydrolysis showed decrease in product inhibition, which can be helpful in improving the industrial performance of the enzyme [55]. 

Alginate-chitosan core-shell microcapsules have also been used for the immobilization of *β*-galactosidase [68]. The rate of 2-nitrophenyl *β*-galactopyranoside conversion to 2-nitrophenol was faster in the case of calcium alginate-chitosan microcapsules as compared to barium alginate-chitosan microcapsules. Barium alginate-chitosan microcapsules, however, did improve the stability of the enzyme at 37°C relative to calcium alginate-chitosan microcapsules or free enzyme. 

Among the three different models (without protection and molecular imprinting technique pretreatment) accomplished for the encapsulation of *β*-galactosidase, the highest enzymatic activity of enzyme was obtained with molecular imprinting technique [114]. The free lactase has been cross-linked into Fe_3_O_4_-chitosan magnetic microspheres for lactulose synthesis by dual-enzymatic method in organic-aqueous two-phase media using lactose and fructose as the raw materials [115]. The organic-aqueous media can significantly promote the transglycosidation activity of lactase and therefore improves the lactulose yield. 

Immobilization technology has shown promising role in reducing the product inhibition of *β*-galactosidase. *A. oryzae* enzyme immobilized on chitosan beads was more effective as compared to nylon membranes to reduce the galactose inhibition [116]. Immobilization of the enzyme on heterofunctional epoxy Sepabeads (boronate-epoxy-Sepabeads and chelate-epoxy-Sepabeads) has shown considerable results in reducing the product inhibition [55]. The effect of internal mass transfer and product (galactose) inhibition on a simulated immobilized enzyme-catalyzed reactor for lactose hydrolysis has been studied [104]. A general mathematical model has been developed for predicting the performance and simulation of a packed-bed immobilized enzyme reactor performing lactose hydrolysis, which follows Michaelis-Menten kinetics with competitive product (galactose) inhibition. The performance characteristics of a packed-bed-immobilized enzyme reactor have been analyzed taking into account the effects of various diffusional phenomena like axial dispersion and internal and external mass transfer limitations. The effects of intraparticle diffusion resistances, external mass transfer, and axial dispersion have been studied and their effects were shown to reduce internal effectiveness factor. *A. oryzae β*-galactosidase was immobilized on the surface of a novel bioaffinity support: concanavalin A layered calcium alginate-starch beads. The immobilized *β*-galactosidase had a much higher *K*_iapp_ value than the free enzyme, which indicated less susceptibility to product inhibition by galactose [98].

## 4. Applications of Immobilized *β*-Galactosidase

Immobilized *β*-galactosidases can be used in a number of ways to hydrolyze lactose in milk, whey/whey permeate, and oligosaccharides synthesis. The choice of process technology depends on the nature of the substrate, the characteristics of the enzyme, economics of production, and marketing of the product. The primary characteristic, which determines the choice and application of a given enzyme, is the operational pH range. Acid-pH enzymes from fungi are suitable for processing of acid whey and whey permeate whereas the neutral-pH enzymes from yeasts and bacteria are suitable for processing of milk and sweet whey.

### 4.1. Hydrolysis of Milk/Whey

Lactose-hydrolyzed milk has been used for the preparation of flavoured milk, cheese, and yoghurt. The hydrolysis of lactose in milk for food processing also prevents lactose crystallization in frozen and condensed milk products. Moreover, the use of hydrolyzed milk in yoghurt and cheese manufacture accelerates the acidification process, because lactose hydrolysis is normally the rate-limiting step of the process, which reduces the set time of yoghurt and accelerates the development of structure and flavour in cheese [1]. The quality of ice milk and ice-cream was significantly improved by addition of lactozyme. It prevents the crystallization of lactose by breaking into glucose and galactose and reduces sandiness [117]. 

High concentration of lactose in whey is a major environmental problem since its disposal in local water streams increases the biological oxygen demand manifolds. The hydrolysis of whey lactose is another important application of *β*-galactosidase in dairy industry. Concentrated hydrolyzed whey or whey permeates can be used as a sweetener in products such as canned fruit syrups and soft drinks [1]. Various immobilizing agents employed for the immobilization of *β*-galactosidase along with hydrolysis of lactose have been summarized in Table 4 [118, 119]. 

Fungal *β*-galactosidase (Miles Chemie) immobilized in polyvinyl alcohol gel was found more thermostable than soluble enzyme, retaining 70% of the activity after 24 h at 50°C and 5% activity at 60°C. A lactose hydrolysis of 75% was obtained in 5-6 h and the degree of conversion decreased to 50% after 30 runs [93]. The studies on the hydrolysis of lactose using immobilized *β*-galactosidase (*Aspergillus niger*) on phenol-formaldehyde resin indicated that the optimal temperature was found to be dependent on the operating time but not on the lactose concentration or the conversion [69]. 

The immobilized *β*-galactosidase from *A. niger* displayed 70% hydrolysis in skim milk at 40°C, with a space time of 10 min [51]. The *β*-galactosidase enzyme of fungal sources immobilized on hydrogels was used for whey hydrolysis and 70%–75% hydrolysis was achieved within 7 h [118]. The immobilization of *β*-galactosidase from *A. oryzae* on activated silica gel resulted in the most active immobilized preparation from TiCl_3_ and FeCl_3_-activated silica gel and resulted in 81 and 84% hydrolysis, respectively, in 4% lactose solution [77]. 

The *β*-galactosidase from *Bacillus circulans* immobilized onto Duolite ES-762 displayed lactose conversion of >70% in a continuous stirred tank reactor [120]. The immobilized *β*-galactosidase from *A. oryzae* in a packed bed reactor displayed 80% of lactose hydrolysis in whey [121] whereas the immobilized *β*-galactosidase from* Saccharomyces fragilis* resulted in a hydrolytic rate of 50% within 3 h in a recycling packed bed reactor [78]. Further, the operational stability was tested, with the system being used up to 5 times before any significant drop in the activity. The addition of Mg^2+^ and Mn^2+^ enhances the hydrolysis of ONPG and lactose by *β*-galactosidase from *K. lactis*, but the rates of activation by each metal on both substrate were not the same [122]. The immobilized *K. lactisβ*-galactosidase from onto CPC-silica (silanizated and activated with glutaraldehyde) and agarose (activated with cyanylating agent) displayed 90% lactose conversion in packed bed minireactors [72]. *β*-galactosidase entrapped in a copolymer gel of *N*-isopropylacrylamide and acrylamide was effective in hydrolysis of lactose at 5°C for production of low lactose milk. It has been observed that lactose conversion decreased the stability of milk casein particles and increased its dispersity [123]. 

 The kinetic model for the lactose using immobilized *β*-galactosidase from *K. fragilis* has also been developed. The immobilized enzyme was active at a low temperature of 5°C and it could also be applied for the production of freeze dairy products to avoid lactose crystallization and to enhance the digestibility and flavour of such products [64]. 

*K. fragilisβ*-galactosidase immobilized on silanized porous glass modified by glutraldehyde binding retained more than 90% of its activity [124]. A lactose saccharification of 86%–90% in whey permeate was achieved both in a batch process and in a recycling packed-bed bioreactor. *K. lactisβ*-galactosidase immobilized onto graphite surface and glutaraldehyde has been used as the cross-linking reagent with the specific activity yield of 17% and 25% while the enzyme loading was 1.8 and 1.1 U/cm^2^ of the graphite external surface area, respectively. It was observed that specific activity yield decreased with the increase of the enzyme loading [113]. Lactose hydrolysis by a *β*-galactosidase from *Thermus *sp. both in solution and immobilized on a commercial silica-alumina was studied [34]. Both the free and the immobilized enzymes are competitively inhibited by galactose, while glucose inhibited only the action of free enzyme, in an uncompetitive way. The immobilization step helped to eliminate the inhibition by glucose. Moreover, the immobilization reduced to a half the inhibitory action of galactose. In general, the immobilization reduced the activity of the enzyme but increased its thermal stability. 

The Lactozym (a commercially available enzyme preparation of *β*-galactosidase obtained from *K. fragilis*) immobilized on cellulose beads has been used for the hydrolysis of whey lactose (>90% conversion) and milk lactose (60% conversion) in 5 h and the immobilized enzyme could be reused three times without any change in the performance of the fluidized bed reactor [14]. The immobilized preparations of *β*-galactosidase from *Thermus* sp. resulted in hydrolysis yield higher than 99%. These immobilized forms of *β*-galactosidase could be used in the total hydrolysis of lactose in milk or dairy whey even at 70°C [55]. The hydrolysis of lactose by immobilized *β*-galactosidase has also been studied in a continuous flow capillary bed reactor by various temperatures. Based on the observed thermal deactivation rate constants, at an operating temperature of 40°C, only 10% of the enzyme activity loss could be there in one year [125]. 

The *β*-galactosidase entrapment in liposomes showed superior thermal stability at various ranges of temperature. Moreover, the proteolytic stability of the *β*-galactosidase was enhanced by encapsulation in liposomes [126]. The entrapment of *β*-galactosidase in liposomes by dehydration-rehydration vesicle method has also been used to prevent an immediate hydrolysis of lactose in milk [96]. *A. oryzaeβ*-galactosidase was immobilized by three different techniques: adsorption on celite, covalent coupling to chitosan, and aggregation by cross-linking and comparing the yield of immobilized preparation, enzymatic characteristics, stability, and efficiency in oligosaccharide synthesis. Cross-linked enzyme aggregates of *β*-galactosidase were found effective in lactose hydrolysis yielding 78% monosaccharide in 12 h [75]. *K. lactisβ*-galactosidase immobilized on cotton fabric using glutaraldehyde as the cross-linking reagent was used for hydrolysis of lactose in whole milk and 95% of lactose conversion has been observed after 2 h of batch operation [23]. 

*K. lactisβ*-galactosidase was covalently immobilized onto a polysiloxane-polyvinyl alcohol magnet, using glutaraldehyde as activating agent that presented a higher operational and thermal stability than the soluble enzyme; so this immobilized *β*-galactosidase was also effectively used for the hydrolysis of lactose from milk [83]. *A. oryzaeβ*-galactosidase was immobilized on silica, the enzyme activity as well as stability has been evaluated, and the best immobilization results were obtained by using glutaraldehyde as support's activator and enzyme stabilizer. Among the different treatments (microfiltration, thermal treatment, and ultrafiltration) of whey, ultrafiltration was the best treatment towards a proper substrate solution for feeding the reactor [84]. 

A recombinant thermostable *β*-galactosidase from *Bacillus stearothermophilus* immobilized onto chitosan using Tris (hydroxymethyl) phosphine (THP) and glutaraldehyde resulted in >80% lactose hydrolysis in milk after 2 h of operation in a packed bed reactor. Thus, THP-immobilized recombinant thermostable *β*-galactosidase from *Bacillus stearothermophilus* has the potential application for the production of lactose-hydrolyzed milk [54]. Calcium alginate entrapped *β*-galactosidase used for the hydrolysis of lactose from solution, milk, and whey in batch processes as well as in continuous packed bed column. It was also observed that entrapped cross-linked concanavalin A-*β*-galactosidase was more efficient in the hydrolysis of lactose present in milk (77%) and whey (86%) in batch processes as compared to the entrapped soluble *β*-galactosidase [93]. Among the two matrices (Sephadex G-75 and chitosan beads) tested for immobilization *β*-galactosidase (BGAL) from *Pisum sativum* for lactose hydrolysis, chitosan-PsBGAL displayed higher rate of lactose hydrolysis in milk and whey at room temperature and 4°C than Sephadex-PsBGA and is better suited for industrial application based on its broad pH and temperature optima, high temperature stability, reusability, and so forth [56]. *β*-galactosidases (from *K. lactis* and *A. oryzae*) were also immobilized in poly(vinylalcohol) hydrogel lens-shaped capsules LentiKats used for the production of D-galactose from lactose (200 g L^−1^) in the batch mode of a simultaneous saccharification and fermentation process [7]. 

*A. oryzaeβ*-galactosidase was immobilized on an inexpensive bioaffinity support, and concanavalin A-cellulose was used for the continuous hydrolysis of lactose from milk and whey. It was observed that the optimum pH for soluble and immobilized *β*-galactosidase is 4.8 but the optimum temperature increased from 50 to 60°C for the immobilized *β*-galactosidase. The immobilized enzyme had higher thermal stability at 60°C [92]. Recently, a packed bed reactor together with alginate entrapped permeabilized cells (*K. marxianus*) has been used for hydrolysis of milk lactose in a continuous system, which resulted in 87.2% hydrolysis of milk lactose [127].

### 4.2. Synthesis of Galacto-Oligosaccharides (GOSs)

Besides hydrolytic action, *β*-galactosidase has also transferase activity by which the enzyme produces and hydrolyses a series of oligosaccharides, which have a beneficial effect on the growth of desirable intestinal microflora [12]. Moreover, the transferase reaction can be used to attach galactose to other chemicals, resulting in formation of galacto-oligosaccharides (GOSs), and consequently have potential application in the production of food ingredients, pharmaceuticals, and other biological active compounds. Nowadays, oligosaccharide production becomes the interesting subject for the researchers, because the oligosaccharides have beneficial effect on human intestinal as “bifidus factor”—promoting growth of desirable intestinal microflora. Oligosaccharides are recognized as useful dietary tools for the modulation of the colonic microflora toward a healthy balance. This usually involves selectively increasing the levels of gut Bifidobacteria and Lactobacilli at the expense of less-desirable organisms such as *Escherichia coli*, *Clostridia*, and proteolytic bacteroides [128]. The amount and nature of oligosaccharides formed depend upon the several factors including the enzyme source, the concentration and nature of the substrate, and reaction conditions [12, 129, 130]. The yield of oligosaccharides can be increased by using higher substrate concentrations or decreasing the water content [31]. 

Although *β*-galactosidase catalyzes both hydrolysis and transgalactosylation reactions, however, the process conditions for lactose hydrolysis and GOS synthesis are different. The reaction conditions for transgalactosylation should be high lactose concentration, elevated temperature, and low water activity in the reaction medium [130]. The temperature, concentration of substrate, and enzyme origin play an important role in the enzymatic synthesis of oligosaccharides [131]. However, the influence of the initial lactose concentration can be much larger [132, 133]. In general, more and larger galacto-oligosaccharides (GOSs) can be produced with higher initial lactose concentrations. The higher temperatures can be beneficial in higher oligosaccharide yields. The higher yield at higher temperatures is an additional advantage when operating at high initial lactose concentrations and consequently elevated temperatures. Hence, immobilized *β*-galactosidase should be stable at high temperature, low water content, and giving high transgalactosylation activity [134]. 

Partially purified *β*-galactosidase from *Bullera singularis* ATCC 24193 immobilized in Chitopearl BCW 3510 bead has been used for the production of galacto-oligosaccharides (GOSs) from lactose in a packed bed reactor, which resulted in 55% (w/w) oligosaccharides with a productivity of 4.4 g/(L-h) during a 15-day operation [132]. The enzyme immobilized on tosylate cotton cloth was used in plug-flow reactor for continuous production of galacto-oligosaccharide from lactose. In general, more and larger GOS can be produced with higher initial lactose concentrations. A maximum GOS production of 27% (w/w) of initial lactose was achieved at 50% lactose conversion with 500 g/L of initial lactose concentration. Tri-saccharides were the major types of GOS formed, accounting for more than 70% of the total GOSs produced in the reactions. The chitosan-immobilized *A. oryzae β*-galactosidase gave maximum trisaccharides yield (17.3% of the total sugar) using 20% (w/v) of lactose, within 2 h as compared to 10% with free enzyme and 4.6% with cross-linked aggregates [73]. 

An immobilized-enzyme system using polyethyleneimine, glutaraldehyde, and cotton cloth was studied and compared the galacto-oligosaccharide production in free-enzyme ultrafiltration and in immobilized-enzyme systems [135]. In the immobilization process, approximately 50% to approximately 90% enzyme inactivation was found with the combination of PEI and GA. Equivalent free- and immobilized-enzyme systems showed very similar maximum GOS production of approximately 22% and approximately 20% (w/v) at approximately 15 to 17 min, 50% conversion for free- and immobilized-systems, respectively. 

The synthesis of galacto-oligosaccharides was optimized with respect to lactose concentration and enzyme to substrate ratio using immobilized *A. oryzaeβ*-galactosidase [136]. In the sequential batch production of galacto-oligosaccharides, biocatalyst efficiency was increased by 190% with respect to the free enzyme in solution, and 8500 g of galacto-oligosaccharides per gram of enzyme preparation were produced after 10 batches. The immobilized *A. oryzaeβ*-galactosidase enzyme on magnetic Fe_3_O_4_–chitosan (Fe_3_O_4_–CS) nanoparticles as support resulted in 15.5% (w/v) maximum yield of galacto-oligosaccharides [137]. The synthesis of galacto-oligosaccharides (GOSs) using *A.* oryzae *β*-galactosidase (free and immobilized) on magnetic polysiloxane-polyvinyl alcohol (mPOS-PVA) has also been carried out [138]. A maximum of 26% (w/v) of total sugars was achieved at near 55% lactose conversion from 50% (w/v) lactose solution at pH 4.5 and 40°C. Trisaccharides accounted for more than 81% of the total GOS produced. GOS formation was not considerably affected by pH and temperature. The concentrations of glucose and galactose encountered near maximum GOS concentration greatly inhibited the reactions and reduced GOS yield.

The packed bed reactor and a plug-flow reactor have been successfully used for continuous production of GOS from lactose using immobilized *β*-galactosidase [63, 90]. The selectivity for GOS synthesis can be increased several-fold under microwave irradiation, using immobilized *β*-glucosidase and with added cosolvents such as hexanol [139]. Recently, a new type of ceramic membrane reactor system using immobilized *β*-galactosidase (*Kluyveromyces lactis*) has been proposed for continuous enzymatic production of galactosyl-oligosaccharides (GOSs) from lactose, which resulted in maximum oligosaccharide (38%, w/w) when the average residence time was 24 min, with an initial 30% (w/w) lactose concentration [140].

## 5. Scale-Up Issues

 For the production of lactose-free milk, the enzyme *β*-galactosidase can be added directly to whole milk, but after complete lactose hydrolysis at a desired level, the enzyme can be deactivated by heat treatment. Since the free enzyme cannot be reused, thus the resulting operation is not cost effective. To overcome this problem, immobilized *β*-galactosidase is used for the hydrolysis of skim milk. After the desired lactose hydrolysis is achieved, cream is added to the hydrolysed milk to adjust its fat content. Although numerous hydrolysis systems have been investigated, only few of them have been scaled up and even fewer have been applied at an industrial or semi-industrial level. 

The first company for the commercial hydrolysis of lactose in milk by using immobilized lactase was Centrale del Latte of Milan, Italy, utilizing the SNAM Progetti technology. The process used an immobilized *Saccharomyces* (*Kluyveromyces*) *lactis* lactase entrapped in cellulose triacetate fibres. Sumitomo Chemical, Japan, has also developed an immobilization process to immobilize *β*-galactosidase of fungal origin on the rugged surface of an amphoteric ion-exchange resin of phenol formaldehyde polymer and this technology was used by Drouin Cooperative Butter Factory for producing market milk and hydrolyzed whey [141]. 

Snow Brand's factory has developed a rotary column reactor that could be used both as a stirred tank reactor and as a packed bed reactor [142]. The reaction rate was greatly affected by the packing density of immobilized *β*-galactosidase in the rotary column. This reactor can also overcome the problem of channeling or severe pressure drop. If the hydrolysis of lactose was carried out in horizontal rotary column, 70%–80% lactose hydrolysis was observed and washing of the immobilized enzyme was carried out for 36 cycles, which indicated that horizontal rotary column reactor was well suited for hydrolyzing lactose in milk with fibrous immobilized enzyme. From the pilot plant experimentations, a commercial plant was set up at Snow Brand's factory [142]. Although the immobilized *β*-galactosidase was washed with phosphate buffer solution and pasteurized with Tego-51, the standard plate count of lactose hydrolyzed milk increased sharply.

Thus, immobilized *β*-galactosidase technology is an effective process for successful hydrolysis of lactose and it can overcome the problems associated with costs of soluble enzyme. However, major problems associated with the immobilized enzyme system are microbial contamination, protein adherence, and channeling. Therefore, for long-term operations using immobilized system, periodic washing, and pasteurization are indispensable processes [143–145]. In immobilized enzyme system, protein adhered to the enzyme can be easily dissolved by using high and low pH solutions, because the immobilized enzyme has high durability over a wide range of pH. The immobilized enzyme can be pasteurized with benzalkonium chloride (quaternary ammonium salt) after removing the proteins. The use of acetic acid solution as a cleaning and pasteurizing agent instead of lactic acid can also be effective. The problem of channeling observed in the packed column system can be overcome by changing the flow direction of feed during the operation [142, 144, 145]. 

Galacto-oligosaccharides are produced simultaneously during lactose hydrolysis due to transgalactosylation activity of *β*-galactosidase. Oligosaccharides/Bifidobacteria provide a wide variety of health benefits, including anticarcinogenic effects, reduction in serum cholesterol, improved liver function, reduction of the colon cancer risk, and improved intestinal health [146, 147]. Therefore, the public demand for their production is significantly increased together with the development of an effective and inexpensive GOS production. Major companies dealing with oligosaccharides production (including GOS) are in Japan [148]. Recently, there is also an increasing trend of GOS production in Europe. Besides lactulose and soybean oligosaccharides, all oligosaccharides are prepared by transglycosylation from mono and disaccharides or by controlled hydrolysis of polysaccharides [147].

## 6. Conclusions

*β*-galactosidase is one of the most important enzymes used in food processing, which offers nutritional, technological, and environmental applications. Enzyme immobilization provides enzyme reutilization and may result in increased activity by providing a more suitable microenvironment for the enzyme. Moreover, immobilized systems can provide better enzyme thermostability and pH tolerance. However, major problems associated with the immobilized enzyme system are microbial contamination, protein adherence, and channeling. The periodic washing and pasteurization and flow direction of feed can solve these problems to great extent. The problem of microbial contamination can also be solved by exploiting the temperature property of the enzyme. The immobilized enzyme preparations showed up to 99% hydrolysis, and thus it can be applied successfully for the hydrolysis of lactose in milk or whey. The isolation of pyschrophilic bacteria with cold active *β*-galactosidase has opened up the possibility of processing of milk and whey even at low temperatures. On the other side, thermostable enzymes have the unique ability to retain their activity at higher temperatures for prolonged periods, and the process is less prone to microbial contamination due to higher operating temperature. Thus, cold active and thermostable enzymes will have the great potential in the lactose hydrolysis and of particular interest to the researchers. Thus, immobilization enzyme systems will certainly find greater role in future times for the hydrolysis of milk, whey, and synthesis of galacto-oligosaccharides.

## Figures and Tables

**Table 1:** Microbial sources of *β*-galactosidase.

Source	Microorganism (s)
Bacteria	*Alicyclobacillus acidocaldarius* subsp. *rittmannii *
	*Arthrobacter *sp.
	*Bacillus acidocaldarius, B. circulans, B. coagulans, B. subtilis, B. megaterum, B. stearothermophilus*
	*Bacteriodes polypragmatus*
	*Bifidobacterium bifidum, B. infantis*
	*Clostridium acetobutylicum, C. thermosulfurogens*
	*Corynebacterium murisepticum*
	*Enterobacter agglomerans, E. cloaceae*
	*Escherichia coli*
	*Klebsiella pneumoniae*
	*Lactobacillus acidophilus, L. bulgaricus,L. helviticus, L. kefiranofaciens, L. lactis, L. sporogenes, L. themophilus, L. delbrueckii*
	*Leuconostoc citrovorum*
	*Pediococcus acidilacti, P. pento*
	*Propioionibacterium shermanii*
	*Pseudomonas fluorescens*
	*Pseudoalteromonas haloplanktis*
	*Streptococcus cremoris, S. lactis, S. thermophius*
	*Sulfolobus solfatarius*
	*Thermoanaerobacter sp.*
	*Thermus rubus, T. aquaticus*
	*Trichoderma reesei*
	*Vibrio cholera*
	*Xanthomonas campestris *

Fungi	*Alternaria alternate, A. palmi*
	*Aspergillus foelidis, A. fonsecaeus, A. fonsecaeus, A. Carbonarius, A. Oryzae*
	*Auerobasidium pullulans*
	*Curvularia inaequalis*
	*Fusarium monilliforme, F. oxysporum*
	*Mucor meihei, M. pusillus*
	*Neurospora crassa*
	*Penicillum canescens, P. chrysogenum, P. expansum*
	*Saccharopolyspora rectivergula*
	*Scopulariapsis sp*
	*Streptomyces violaceus*

Yeast	*Bullera singularis*
	*Candida pseudotropicalis*
	*Saccharomyces anamensis, S. lactis, S. fragilis*
	*Kluyveromyces bulgaricus, K. fragilis, K. lactis, K. marxianus*

Source: [12–26].

**Table 2:** Different sources of *β*-galactosidase and methods of immobilization.

Immobilization method	Source of *β*-galactosidase	Immobilizing agents	References
(1) Physical adsorption	*K. fragilis and K. lactis*	Chitosan	[6]
	*A. oryzae*	Phenol-formaldehyde resin	[69]
	*A. oryzae*	Polyvinyl chloride and Silica gel membrane	[51]
	*E. coli*	Chromosorb-W	[52]
	*B. circulans*	Polyvinyl chloride and Silica	[53]
	*B. stearothermophilus*	Chitosan	[54]
	*A. niger*	Porous ceramic monolith	[70]
	*K. fragilis*	Chitosan bead	[2]
	*K. fragilis*	Chitosan	[71]
	*K. lactis*	CPC-silica and agarose	[72]
	*Thermus* sp. T2	PEI- sepabeads, DEAE-agarose	[55]
	*K. fragilis*	Cellulose beads	[14]
	*A. oryzae*	Celite and chitosan	[73]
	*Pisum sativum*	Sephadex G-75 and chitosan beads	[56]

(2) Entrapment	*K. bulgaricus*	Alginate using BaCl_3_	[57]
	*E. coli*	Polyacrylamide gel	[58]
	*A. oryzae*	Nylon-6 and zeolite	[62]
	*Thermus aquaticus* YT-1	Agarose bead	[59]
	*A. oryza*	Spongy polyvinyl alcohol Cryogel	[60]
	*Penicillium expansum* F3	Calcium alginate	[23]
	*K. lactis*, *A. oryzae Saccharomyces cerevisiae *	Poly(vinylalcohol) hydrogel	[7]

(3) Covalent Binding	*L. bulgaricus*	Egg shells	[61]
	*S. anamensis*	Calcium alginate	[74]
	*E. coli*	Hen egg white	[75]
	*E. coli*	Polyvinyl alcohol	[76]
	*A. oryzae*	Silica gel activated with TiCl_3_ and FeCl_3_	[77]
	*E. coli *(Recombinant *β*-galactosidase)	Cyanuric chloride-activated cellulose	[66]
	*K. lactis*	Corn grits	[78]
	*E. coli*	Gelatin	[63]
	*K. lactis*	Thiosulfinate/thiosulfonate	[79]
	*B. circulans*	Eupergit C (Spherical acrylic polymer)	[65]
	*K. fragilis*	Silica-alumina	[64]
	*K. lactis*	Graphite surface	[68]
	*A. oryzae*	Chitosan bead and nylon membrane	[80]
	*A. oryzae*	Cotton cloth and activated	[81]
		With tosyl chloride	
	*A. oryzae*	Amino-epoxy sepabead	[67]
	*K. latis*	Cotton fabric	[82]
	*A. niger*	Magnetic polysiloxane-polyvinyl alcohol	[83]
	* A. oryzae*	Silica	[84]
	*A. oryzae*	Polyvinylalcoheol hydrogel and magnetic Fe_3_O_4_-chitosan as supporting agent	[85]

**Table 3:** Cross-linking reagents used in *β*-galactosidase immobilization.

Cross-linking reagent	References
Bis-oxirane	[102]
Carbodiimide	[103]
Chromium (III) acetate	[63]
Glutaraldehyde	[14, 20, 69, 70, 104–107]
Polyethyleneimine	[57, 101, 108]
Sulfate-dextran	[109]
Transglutaminase	[110]
Tris(hydroxymethyl)phosphine	[54]

**Table 4:** Hydrolysis of lactose with various immobilizing techniques of *β*-galactosidase.

Microbial source	Immobilizing agent	% Lactose hydrolysis	Time of hydrolysis	References
Fungal galactosidase (Miles Chemie)	Polyvinyl-alcohol	75%	5-6 h	[93]
*E. coli*	Polyacrylamide gel	47%	6 h	[58]
*K. lactis*	Thiosulfinate/thiosulfonae	85%–90%	2.5 h	[79]
*K. fragilis*	Cellulose beads	>90%	5 h	[14]
*K. lactis*	Cotton fabric	95%	2 h	[82]
Fungal *β*-galactosidase	Hydrogels	70%–75%	7 h	[116]
*K. marxianus*	Calcium alginate	84.8%	2.5 h	[117]
*A. oryzae*	Concanavalin A layered calcium alginate-starch hybrid beads	89%	3 h	[98]
*Bacillus stearothermophilus*	Chitosan	>80%	2 h	[54]
